# Comparative evaluation of comprehensive offline 2D-LC strategies coupled to MS for untargeted metabolomic studies of human urine

**DOI:** 10.1007/s00216-025-06195-2

**Published:** 2025-11-01

**Authors:** Maria Grübner, Andreas Dunkel, Frank Steiner, Thomas Hofmann

**Affiliations:** 1 Chair of Food Chemistry and Molecular Sensory Science, Technical University of Munich, Lise-Meitner-Straße 34, 85354 Freising, Germany; 2 Thermo Fisher Scientific, Dornierstraße 4, 82110 Germering, Germany; 3 Institute of Physiological Chemistry, Faculty of Chemistry, University of Vienna, Josef-Holaubek-Platz 2, 1090 Vienna, Austria

**Keywords:** Comprehensive offline two-dimensional LC, Metabolomics, Mixed-mode chromatography, HILIC, Fractionation, Evaporation

## Abstract

Out of the broad selection of analytical methods applied in metabolomic studies, liquid chromatography coupled to mass spectrometry (LC-MS) has the highest coverage potential. In that regard, the quality of the separation process is crucial for the analytical outcome. Reversed-phase (RP) and hydrophilic interaction liquid chromatography (HILIC) are widely applied, and the potential of various setups to combine these modes for more complementary data has been deeply explored. In our previous study on the orthogonality of LC conditions in the field of metabolomics, combinations of a mixed-mode phase with parallel RP and ion-exchange (IEX) properties and several HILIC columns exhibited the widest compound distributions in a two-dimensional (2D) separation space. For further performance evaluation, an offline comprehensive 2D-LC-TOF-MS (LC×LC-TOF-MS) system was set up with the mixed RP/IEX mode in the first dimension (^1^D) and HILIC mode in the second dimension (^2^D). The transfer of fractions to the HILIC column and the effect of offline fraction preparation procedures (dilution and evaporation approaches) were comparatively investigated by using reference substances. In addition, the separation performance of the offline LC×LC-TOF-MS system with and without offline fraction preparation was assessed in comparison to other common LC-TOF-MS strategies (direct flow injection DFI, 1D-LC, serial coupling LC) by the number of detectable features in a human urine sample. In conclusion, the direct transfer of 5 µL fraction volumes without offline treatment was the most promising approach for future application in untargeted metabolomic studies for marker identification from human urine.

**Supplementary Information:**

The online version contains supplementary material available at 10.1007/s00216-025-06195-2.

## Introduction

Metabolomics, the study of low molecular weight compounds in biological systems, includes the profiling of microbiological, plant, and mammalian material to serve in various fields like disease diagnostics, drug development, and toxicology [1, 2].

Universal resolution and detection by a single analytical technique is not realistic due to the sheer number and structural diversity of relevant components. As a ballpark, the human metabolome database currently lists > 220.000 entries, but metabolome size may reach ~ 5 million compounds [3, 4]. Thus, the demand for methods as comprehensive as possible is huge [2, 5].

From the numerous analytical strategies applied, liquid chromatography (LC) coupled to mass spectrometry (MS) stands out regarding its potential chemical space coverage. Recent estimates suggest that up to 85% of the known human metabolome could be covered by LC-MS, making it the key strategy in metabolomics [4, 6, 7]. Despite substantial advances in MS technology, the chromatographic separation is crucial to enhance sensitivity and selectivity by reducing ion suppression and interferences of isomeric compounds and fragments [5, 7]. Hence, the analytical outcome severely depends on the LC separation applied [1, 8]. RP chromatography is widely used in metabolomic studies due to its high efficiency, reproducibility, and selectivity in the separation of unpolar and medium polar components [7]. However, only 5–10% [4] of the detected metabolome consists of unpolar compounds, meaning that the predominant polar portion mostly elutes in the void volume and remains undetected due to ion suppression [4, 8, 9]. Ion-pairing (IP) reagents can address retention limitations but may compromise MS detection [7, 10]. Hence, HILIC is preferred in retaining polar metabolites [1, 8, 9]. Simultaneous retention of hydrophobic and hydrophilic metabolites can be targeted by mixed-mode stationary phases [11]. Still, no single chromatographic mode can cover the huge polarity range of metabolites [5, 7]. Consequently, the combination or hyphenation of analytical techniques is frequently implemented to increase metabolome coverage [2, 5, 6, 10].

Recently, we reported on the extended distribution of urine metabolites in the separation space of a comprehensive 2D-LC (LC×LC) method, including a mixed-mode RP/IEX and a HILIC dimension [12]. The efficiency of RP/HILIC combinations has been proven earlier in numerous instrumental setups [5, 8, 10]. Simple dual separations provided complementary data to enhance biomarker detection for liver diseases [13], and parallel column setups increased throughput in primary metabolite quantification in yeast cell extracts [14]. With column-switching setups, samples are split into HILIC and RP retained parts and successively eluted to increase feature counts [15]. Serial RP-HILIC coupling follows a similar concept. The connection between the columns is implemented by a T-piece to feed a make-up gradient for the second column with gradient profiles aiming either for consecutive or overlapping elution of both columns [16, 17]. Effective retention and separation of metabolites extracted from beer have been demonstrated [17]. Directly in tandem coupled columns, eluted with a single gradient, yielded 20–30% more MS-detected features from mouse serum than a single column [18].

While few strategies might be interpreted as heart-cut 2D-LC (LC-LC), 2D-LC application generally takes off slowly in metabolomics, despite the enormous potential of especially LC×LC—the technique submitting the entire sample to both dimensions [19]. Publications mainly focus on distinct targets or metabolomic subsections (lipids, phenols) [19].

High-throughput profiling of 141 water-soluble microorganism metabolites was established by offline strong cation exchange (SCX)×HILIC [20]. Urine samples and cell cultures were screened by RP×RP for modified nucleosides as potential breast cancer markers [21]. Enantioselective analysis of, e.g., amino acids was achieved by heart-cutting to chiral columns in the ^2^D [22]. HILIC×RP combinations proved effective in lipid [23] and phospholipid profiling [24], with HILIC separating lipid classes and polar head groups, and RP separating individual species by fatty acid composition [19]. Global metabolic profiling by LC×LC is less common. Polar retention and orthogonality in human urine profiling by offline RP×RP were achieved by IP and derivatization, triplicating detectable MS features compared to 1D-LC [25]. An untargeted HILIC×RP-MS study investigated environmental effects on the rice metabolome [26], and another one the metabolite profiles of licorice [27]. More comprehensive overviews are provided in some recent reviews [7, 19, 22, 28].

HILIC×RP or RP×HILIC seem promising in increasing metabolome coverage [2]. However, recently, we found them suboptimal combinations, with the majority of tested urine compounds being retained by only one mode, resulting in moderate orthogonality [12]. An RP/IEX×HILIC-high-resolution MS (HRMS) method demonstrated outstanding orthogonality and potential for maximizing coverage, verified by targeted screening of urine compounds [12]. The efficiency of this system in untargeted metabolomic profiling studies needs further characterization. To the best of our knowledge, it is the first time an RP/IEX×HILIC-HRMS system has been investigated regarding its untargeted separation and detection capabilities of human urine components.

## Experimental section

### Materials and reagents

For mobile phase preparation, water was purified by means of a Milli-Q Advantage A10 water purification system (Millipore, Molsheim, France). Acetonitrile (ACN, LC-MS grade) and ammonium acetate solution (NH_4_Ac, 5 M) were obtained from Sigma-Aldrich (Steinheim, Germany). Acetic acid was purchased from Merck KGaA (Darmstadt, Germany). The standard compounds L-alanine, L-proline, L-leucine, L-glutamic acid, L-phenylalanine, L-tyrosine, L-tryptophan, L-ornithine monohydrochloride, choline chloride, acetylcholine chloride, urocanic acid, hippuric acid, melatonin, β-(-)-adenosine, testosterone, (-)-riboflavin, L-carnitine hydrochloride, O-acetyl-L-carnitine hydrochloride, palmitoyl-DL-carnitine chloride, adenosine 5′-monophosphate monohydrate, 5-aminovaleric acid hydrochloride, putrescine dihydrochloride, N-acetyl-L-aspartic acid, creatine, sarcosine, taurine, D-mannitol, D-pantothenic acid calcium salt, serotonin hydrochloride, inosine, allantoin, 1,3-dimethyluric acid, 7-methylxanthine, and uracil were obtained from Sigma-Aldrich (Steinheim, Germany). Caffeine was purchased from Merck KGaA (Darmstadt, Germany), rac octanoyl carnitine chloride from Toronto Research Chemicals (Toronto, Canada), 2-phenylacetamide from TCI chemicals (Tokyo, Japan), D(+)-biotin from Carl Roth (Karlsruhe, Germany), lactose monohydrate from VWR International BVBA (Leuven, Belgium), and trigonelline from Extrasynthese (Genay, France).

### Samples

For HILIC injection experiments, a compound mixture covering the HILIC gradient (given in the supplementary information SI-1) was prepared with analyte concentrations of 10–20 mg/L in ACN/water compositions ranging from 0 to 100% water in 10% steps. Another test mixture covering the RP/IEX gradient (see SI-1) was prepared in water/ACN (1/1, v/v) at concentrations of ~ 100 mg/L for fraction treatment evaluation. Pooled lyophilized urine samples were obtained from a study conducted by Lang et al. [29] in our institute. They were reconstituted in water to the original concentration to compare untargeted LC-MS strategies.

### Instrumentation

**Fig. 1:**
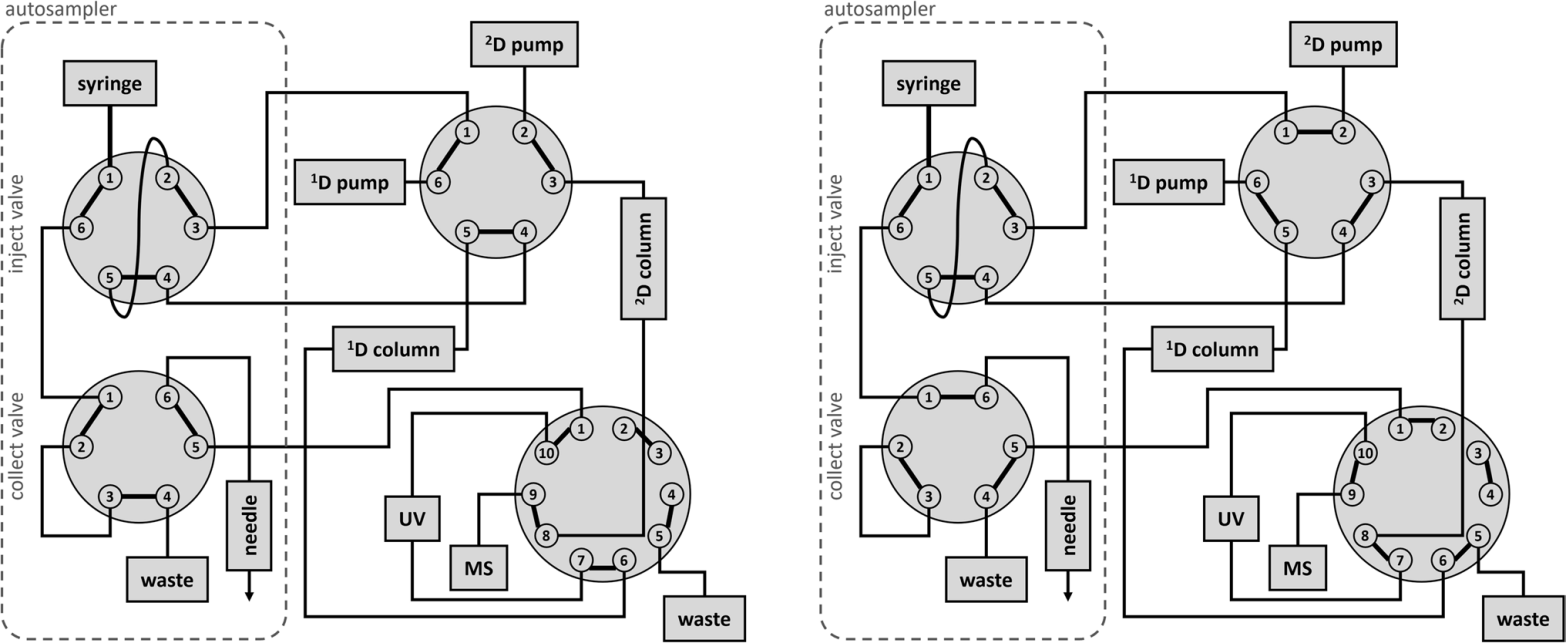
Automated offline 2D-LC setup scheme. The injection and fraction collection valves are located in the autosampler, which serves as injector and fraction collector. Left: For the ^1^D separation, the 6-port valve is in 6_1 position and the 10-port valve in 10_1 position. ^1^D eluate is monitored by UV detection and sampled into vials. Right: Both valves are switched to 1_2 position for fraction reinjection and ^2^D separation with subsequent MS detection

The offline 2D-LC instrument was an Ultimate 3000 UHPLC system (Thermo Fisher Scientific, Germering, Germany) equipped with an autosampler with fraction collection capabilities (WPS-3000TFC), a dual gradient pump (DGP-3600RS) with degasser (SRD-3600), a column compartment (TCC-3000RS), a UV detector (VWD-3400RS), and a 6-port 2-position valve and a 10-port 2-position valve for 2D-flow arrangement (see Fig. 1). For offline LC×LC-MS/MS analysis of a test mixture, the system was connected to an API 3200 triple quadrupole mass spectrometer (AB Sciex, Darmstadt, Germany); for offline LC×LC-TOF-MS analysis of urine, the ^2^D was hyphenated to a TripleTOF 6600 system (AB Sciex). The instrument was controlled by Chromeleon CDS (version 6.8, Thermo Fisher Scientific) and Analyst 1.5.1 (AB Sciex), respectively, Analyst TF 1.7.1 software (AB Sciex). Experiments in 1D mode were conducted with the setup of the ^2^D of the 2D-LC instrument. For HILIC injection experiments, the column was coupled to a Bruker microTOF-Q mass spectrometer (Bruker Daltonics, Bremen, Germany) controlled by DataAnalysis version 3.4.

Serial coupling experiments based on the configuration of Greco et al. [16] were modified to prevent 1st column damage due to 2nd column back pressure. Pump 1 and column 1 were similarly connected via the 6-port and injection valve, as shown in Fig. 1. Column 1 eluate was then directed to an adjustable flow splitter (Analytical Scientific Instruments, Richmond, CA, USA). The reduced flow was fed into a Nexera X2 LC-30AD pump (Shimadzu, Duisburg, Germany), which pumped the incoming flow in an isocratic setting to a T-piece, where the make-up flow for column 2 was added by pump 2. The third port of the T-piece was connected to column 2, which released the eluate into the TripleTOF 6600 spectrometer. The setup is shown in the supplementary information SI-2.

### Chromatographic conditions

In this study, one mixed-mode and one HILIC column were used. The Acclaim Trinity P1 column (50 × 2.1 mm, 3 µm, Thermo Fisher Scientific, Sunnyvale, USA) was operated in mixed RP and IEX mode under ternary gradient conditions with mobile phase: (A) acetonitrile, (B) water, (C) 200 mM NH_4_Ac in water pH 3.8. Two gradient conditions, MM-1 and MM-2, were utilized as given in the supplementary information SI-3. The Accucore-150-Amide-HILIC column (150 × 2.1 mm, 2.6 µm, Thermo Fisher Scientific, Sunnyvale, USA) was operated at 0.4 mL/min under gradient conditions with mobile phase: (A) ACN, (B) water, (C) 100 mM NH_4_Ac in water pH 4.3. Four different gradients (H-1, H-2, H-3, H-4) were used, given in SI-3. MM-1, MM-2, H-1, H-2, and H-3 gradients were either adopted or slightly adjusted from our previous work [12]; H-4 was specifically for the serial column coupling experiments. The pH of mobile phases C was adjusted by the addition of acetic acid after the respective dilution of the commercial NH_4_Ac stock solution. While the absolute NH_4_Ac concentration in the HILIC gradients H-1, H-2, and H-3 was kept constant at 10 mM (10% C) for MS compatibility, the NH_4_Ac concentration in the ternary MM gradients ranged from 5 to 50 mM (2.5 to 25% C) for the IEX elution, which was not considered suitable for MS infusion. The column oven temperature was 40 °C, and the autosampler temperature was 4 °C at any time.

### HILIC injection experiments

Experiments on suitable injection volumes for the ^2^D HILIC column were conducted with an analyte mixture covering gradient H-1. Volumes from 0.5 to 10 µL were injected in 11 different ACN/water compositions on the HILIC column. The microTOF-Q was operated in full scan positive ESI mode (see SI-3).

### Targeted fraction treatment comparison

**Fig. 2:**
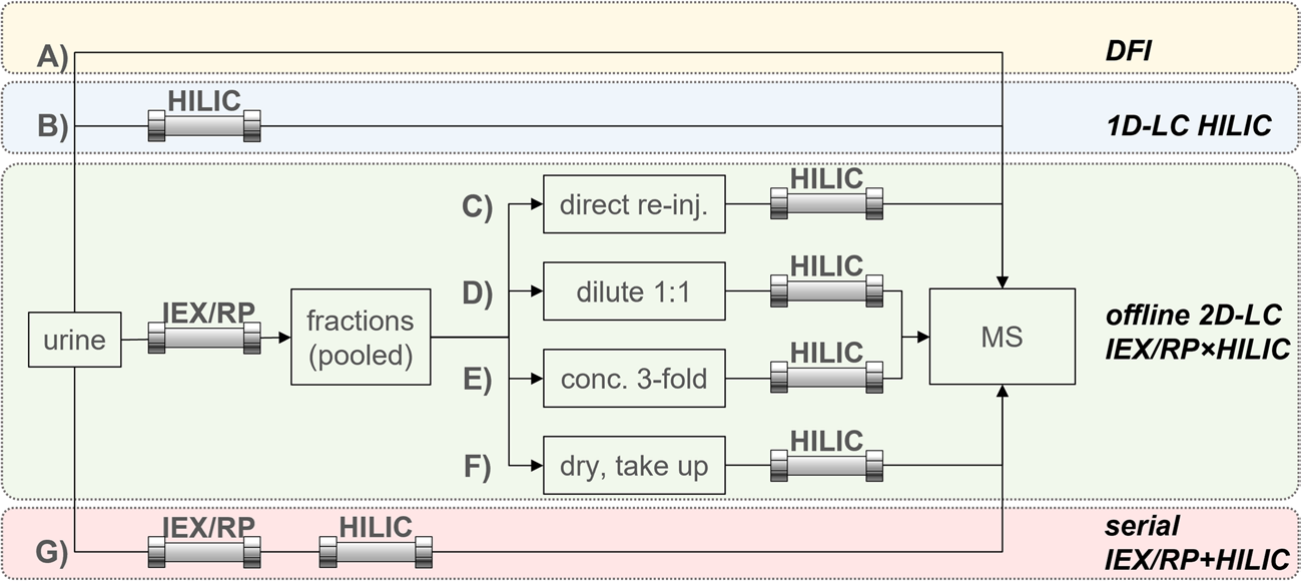
Illustration of comparison experiments for LC-MS strategies (detailed conditions given in the text)

Different fraction preparation procedures were compared in offline 2D-LC (C–F in Fig. 2) and were conducted by targeted MS/MS analysis of a test mixture. Ten microliters of test solution was injected and fractionated by gradient MM-1 with the mixed-mode column in the ^1^D. Fractions were collected from 0.5 to 13 min every 30 s, pooled over 13 injections, and divided into 4 aliquots for manual preparation. For procedures C, D, E, and F in Fig. 2, one aliquot did not undergo any treatment, one was diluted with ACN (1/1; v/v), one was evaporated under a nitrogen stream to one-third of its initial volume, and one was evaporated to dryness and resolved in one-third of its initial volume in ACN/water (1/1; v/v). Injection volumes for ^2^D were 4 µL, except for diluted aliquots (10 µL). ^2^D separation was performed on a HILIC column with gradient H-2. The API 3200 was operated in positive ESI and multiple reaction monitoring (MRM) mode (see SI-3).

### Comparison of untargeted LC strategies

An untargeted approach of comparing direct flow injection (DFI), 1D HILIC separation, and 2D-LC with different fraction treatments and serial coupling of both columns was conducted with a pooled urine sample (see Fig. 2). Five microliters of urine was (A) directly injected into a flow of 0.2 mL/min (50% water, 50% ACN) without separation, (B) separated by a HILIC method (gradient H-3), and (C–F) separated and fractionated by gradient MM-2 on the mixed-mode column. Fractions were collected from 0.5 to 25.5 min every 30 s, pooled over five injections, and manually processed as above. Reinjection of 5 µL (10 µL for diluted aliquots) was conducted by gradient H-3 on the HILIC column. Finally, for approach G, 5 µL of urine was separated on mixed-mode and HILIC columns connected in series. The mixed-mode gradient was MM-2 with the final composition held for 2 more minutes, the flow was split to 0.05 µL/min, and the mobile phase of the HILIC column was a composition of this split eluate and gradient H-4. All experiments were recorded in positive and negative ESI mode with high sensitivity setting by the TripleTOF spectrometer with an initial TOF-MS full scan followed by information-dependent (IDA) product ion acquisition in a range of 50–1000 m/z (see SI-3).

### Data processing

LC-MS raw data files were converted to the mzML format by the MSConvert tool (version 3.0.20321-6df943caa) from the ProteoWizard [30] toolkit. Subsequent pre-processing was performed using the xcms package (3.22.0) [31–33] within the R programming platform (version 4.3.2). In detail, in a first step, peaks were detected in the individual ^2^D runs by means of the centWave algorithm with the following settings: snthresh 10, ppm 30, prefilter (5, 1000), peakwidth 5–30. Following retention time adjustment using the obiwarp [34] method, peaks were grouped within corresponding ^1^D fractions (bw 30, minFraction 0). In a final step, peaks detected in ^2^D runs of adjacent ^1^D fractions were merged to yield individual features. Heatmaps were created using the pheatmap package (version 1.0.12) [35] within R, and the ggVennDiagram (version 1.5.2) [36] package for Venn diagrams.

## Results and discussion

**Fig. 3:**
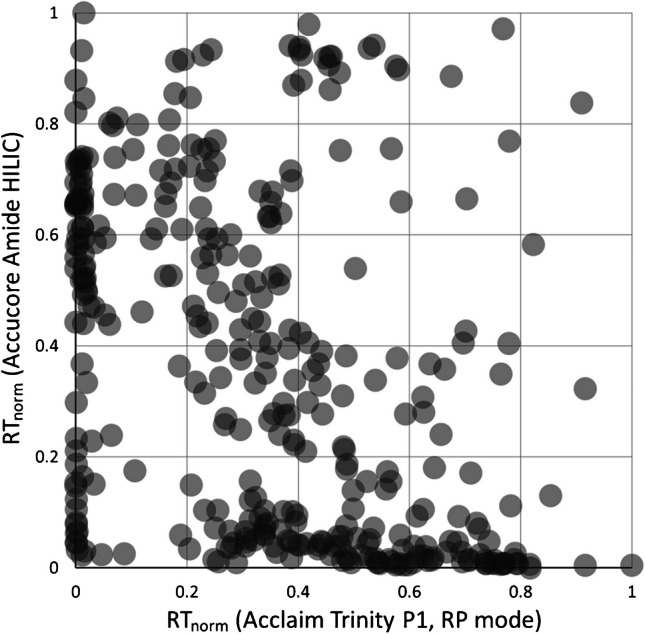
Theoretical distribution of metabolites in a 2D separation space provided by the conditions employed in the current study. Detailed information was published elsewhere [12]

Recently, we reported our efforts in finding convenient column combinations for LC×LC systems for application in metabolomic investigations based on retention time sets of several hundred metabolites listed in the Human Metabolome Database [3, 12]. Herein, most orthogonal data distributions were obtained by combining a mixed-mode RP/IEX column with HILIC columns. Figure 3 illustrates the respective data set for the columns and gradients selected for the subsequent experiments. This study targets the evaluation of the proposed LC×LC-MS system, comprising the Acclaim Trinity P1 mixed-mode and the Accucore-150-Amide-HILIC column, regarding its potential in untargeted compound detection. The assignment of the ^1^D and ^2^D columns is dictated by the high (volatile) salt concentration of the mixed-mode mobile phase, which makes the mixed-mode column ineligible for the ^2^D with direct MS coupling. Hence, HILIC is applied in the ^2^D. In this study, offline fraction transfer is considered more appropriate over the predominant online approaches [28]. It is the way to go when ^2^D cycle times cannot be easily reduced to the ^1^D fraction time without losing essential contributions to the overall resolution, which applies to our HILIC method. With this, the effect of fraction preparation steps between the chromatographic dimensions in targeted and untargeted approaches also becomes of particular interest. Several fraction treatments are investigated and are furthermore compared to three additional common LC-MS strategies.

### Effect of injected water amount in HILIC chromatography

**Fig. 4:**
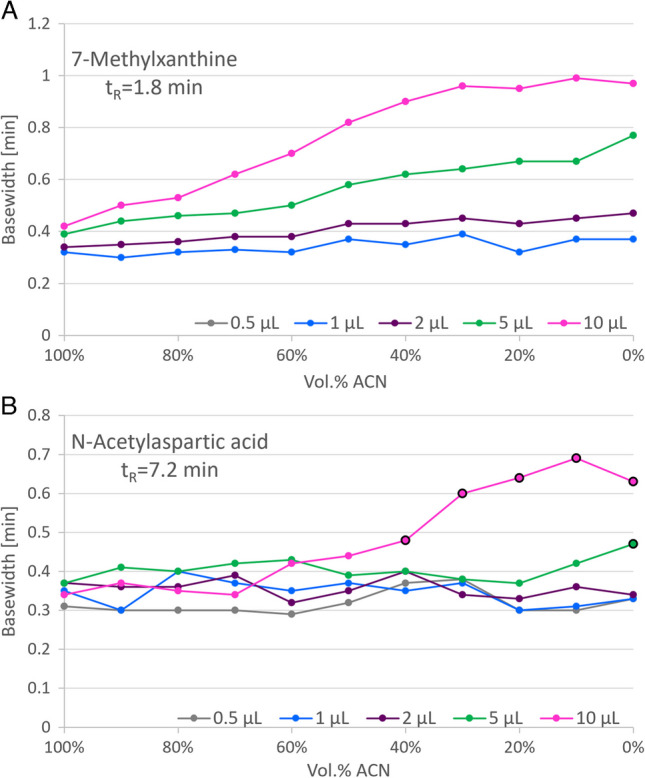
Example plots for the effect of injected volume and water proportion in the HILIC dimension. **A** Base width of early eluting 7-methylxanthine increased with injection volume and water amount; 0.5 µL injections were not detected. **B** Base width of late eluting N-acetylaspartic acid was hardly affected by injection volume and only significantly increased with a 10 µL injection and ≤ 40% ACN, also combined with peak splitting (marked by circled data points)

As the elution strengths of water and ACN are inverted in RP and HILIC chromatography, online coupling of these modes in LC×LC is usually not straightforward [37]. Solvent strength mismatch effects may affect analyte retention, peak shape, and sensitivity. The same is true for the applied RP/IEX and HILIC conditions, which additionally contributed to the decision to apply an offline fraction transfer from the ^1^D to the ^2^D. To specify a proper reinjection volume (0.5–10 µL) of fractions into the HILIC dimension, avoiding severe peak deterioration due to injection effects, peak widths were evaluated as a function of the water proportion of the injection plug for standard compounds covering the HILIC gradient. The effect of injection volume and water amount in the injection solvent on peak width was not consistent for the individual components. However, it is not surprising that the general trend showed that late eluting analytes were less affected by increasing water proportions in the injection plug as they required higher water contents for elution. On the other hand, compounds eluting earlier in the gradient were more sensitive to that proportion, particularly when the injection volume was 5 or 10 µL. The effect of high water amounts combined with increasing injection volume ranged from loss of retention and peak broadening to peak distortion and peak splitting. Exemplary peak width plots are given in Fig. 4. For most of the test compounds, acceptable peak shapes and widths were obtained with the injection of 5 µL even at higher water contents. Thus, this volume was selected as the best compromise for fraction reinjection. It corresponded to 1.5% of the void volume of the ^2^D HILIC column, while in other studies, up to 9% were reported [37]. Retention times, of course, were also affected by the water proportion of the injection plug. Again, early eluting compounds were affected most, especially at the 5 and 10 µL stage, while late eluting metabolites were hardly impacted.

### Targeted comparison of fraction preparation procedures

One benefit of offline 2D-LC setups over online approaches is their potential to integrate fraction preparation steps, like concentration, dilution, or solvent exchange, into the process, with the objective of enhancing the detectability of analytes or compatibility of the dimensions. Recent applications can be found in the field of metabolomics and other studies, often with HILIC in the ^2^D [28, 38, 39]. As the injection of only a few microliters from fractions of 100–150 µL volume in our study is equivalent to a further dilution, fraction treatments appeared to be worthwhile to examine in terms of sensitivity enhancement but also in terms of potential risks like contamination or chemical stability.

**Fig. 5:**
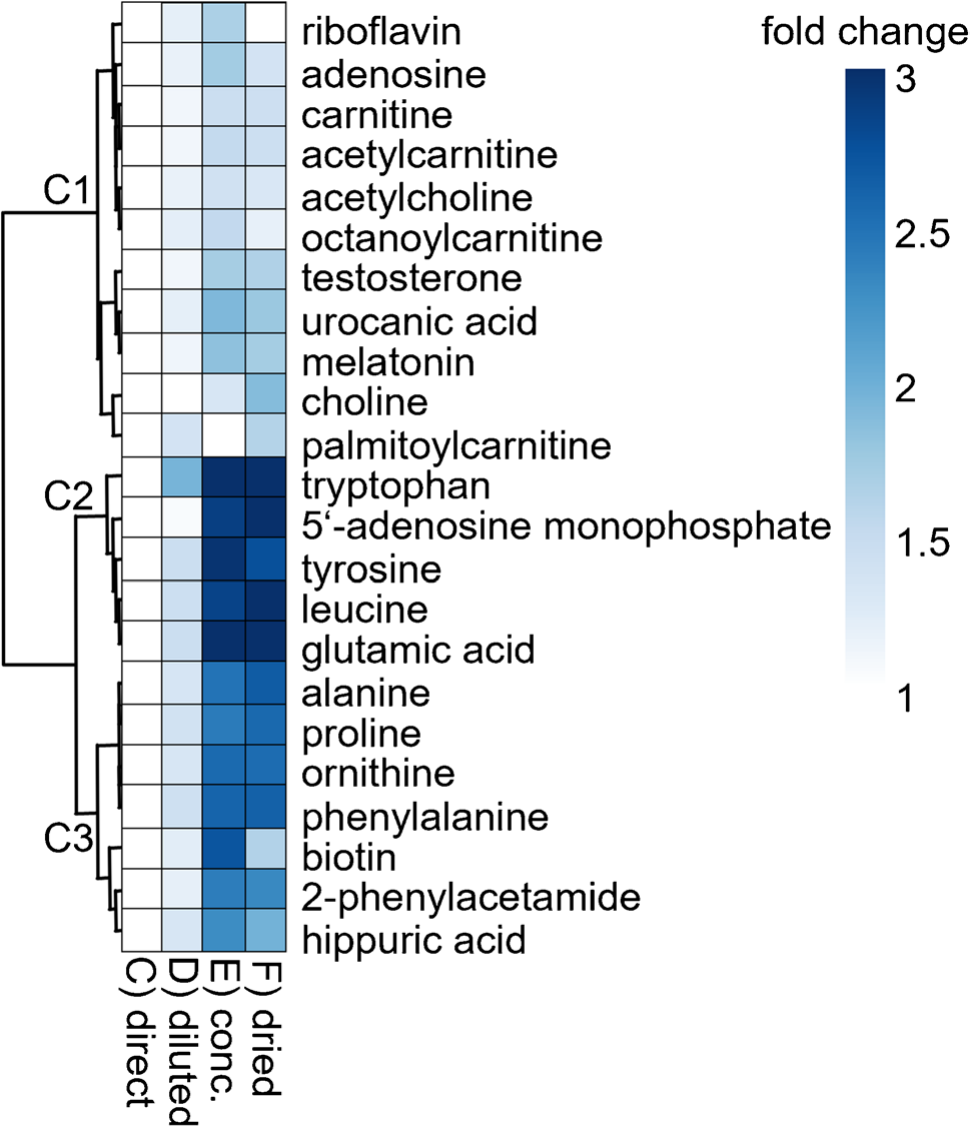
Heatmap of peak area ratios—normalized to a direct reinjection with no fraction treatment—for 23 targeted analytes and different fraction treatments

The reinjections of differently processed ^1^D fractions were compared by targeted LC-MS/MS using peak area relations of 23 test compounds distributed over the ^1^D gradient. Treatments, which were evaluated in comparison to a direct reinjection C, included (D) dilution (1/1; v/v) with ACN and reinjection of 2.5-fold volume, (E) reduction to one-third of volume, and (F) complete evaporation of fraction solvent followed by take up in one-third of initial volume in a uniform solvent mixture of ACN/water (1/1; v/v). The resulting peak area ratios are summarized in Fig. 5, and the supporting material SI-4 (Table SI-1). On average, peak areas 1.27, 2.33, and 2.28 times larger than in untreated fractions were found by procedures D, E, and F. Achieving peak area increments by diluting fractions in weak eluent is in good agreement with Stoll et al. [40]. These were quite homogenous, ranging from 1.01 to 1.44 for all compounds except tryptophan (1.93) as apparent from the relatively smooth color distribution for treatment D in Fig. 5. However, the ratios in concentrated and dried fractions were far more variable, ranging from 0.47 to 6.3 and from 0.96 to 5.91, marked by very light and dark colors in Fig. 5. Values < 1 indicated some drawbacks of preparations entailing evaporation, possibly causing analyte loss by degradation due to pH effects, incomplete solubility, or adhesion to the vessel walls. No correlation was found between the occurrence of very high or very low area ratios and the fraction numbers/^1^D retention, which gives rise to the assumption that it is more related to the nature of the particular analyte than to the specific solvent conditions of a fraction. This assumption is supported by the formation of three compound clusters in Fig. 5, showing similar tendencies for treatments E and F (light, cluster C1; medium, cluster C2; and dark shadings, cluster C3).

From a quantitative point of view, the average sensitivity increase obtained by fraction preparation did not result in very appealing levels, particularly if the time and labor for manual evaporation were taken into account. Gentle volume reduction to less than one-third is feasible but more challenging, especially for highly aqueous fraction solvents. Some pre-experiments for automating the offline evaporation by feeding a nitrogen stream through the autosampler needle resulted in long preparation times (data not shown). This simple approach would not have supported the preferred workflow of parallel ^2^D analysis and processing of the subsequent fraction, and thus was not followed up. In addition, the evaporation reproducibility was assessed as critical but hard to verify. Some more sophisticated attempts targeting online evaporation have been published. These employed vacuum or heating coils with optical control and were developed for the evaporation of volatile NP mobile phases or the hyphenation of LC and NMR spectroscopy [41, 42]. Evaporation through a porous hydrophobic membrane modulator was evaluated for a test mix in RP×RP [43]. However, all mentioned techniques required significantly more complex equipment. Intriguing work has also been published on sensitivity enhancement by focusing and thus online concentration by trap columns [44, 45], temperature modulation [45, 46], or dilution flows of weak eluents [40]. In online LC×LC, the active modulation of the fraction solvent by pre-mixing with the ^2^D flow is very effective [28]. Most techniques focus on RP columns in the ^2^D [47]. For online RP×HILIC combinations, an approach employing a third flow for fraction transfer and focusing has been showcased [48]. A transfer to our offline approach would indicate the need for extended hardware and fluidic complexity.

### Comparison of untargeted LC–MS strategies

**Fig. 6:**
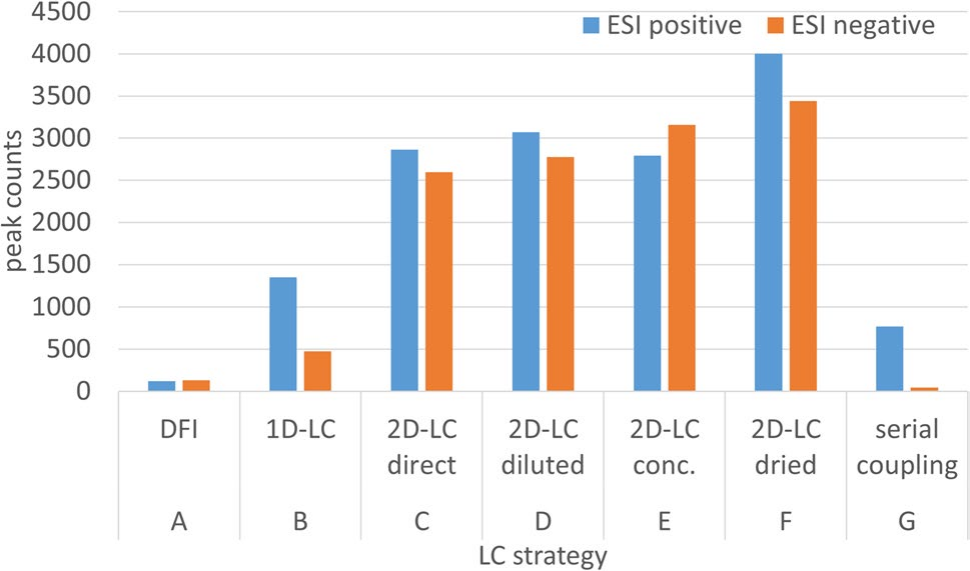
Number of individual features detected by (A) DFI, (B) HILIC separation, (C-F) offline 2D RP/IEX×HILIC separations with and without fraction treatment as outlined in the text and (G) separation by serially connected RP/IEX and HILIC columns coupled with TOF–MS

While the sensitivity increase in targeted analysis by fraction treatment in 2D-LC was moderate, a more critical aspect was its effect on the analytical information gained from untargeted analysis of actual samples. Thus, offline 2D-LC with the different fraction treatments as above, as well as other common LC techniques based on the same column hardware, was compared in terms of detectable individual feature counts of the same urine sample when coupled to TOF-MS. The experiments included (A) DFI without any separation, (B) HILIC separation, (C–F) offline RP/IEX×HILIC separations with and without fraction treatment as above, and (G) separation by serially connected RP/IEX and HILIC columns (Fig. 2). A parallel dual-column approach was omitted for the comparison due to the MS incompatibility of the mixed-mode mobile phase. Peak counts traced in positive and negative ESI-TOF-MS are summarized in Fig. 6 (see also SI-5). The peak detection criteria applied for the LC–MS strategies were selected rather conservatively to avoid overestimation. Correction for peaks found in blank injections was applied, as well as alignment of features in adjacent 2D-LC fractions.

The 2D-LC approach C with direct reinjection achieved counts of 2864/2598 features in positive/negative ESI mode. These numbers were more than double/five-fold compared to the 1D-HILIC separation B (1350/476). As expected, the lowest numbers were achieved with the DFI approach A (119/134), with less than a tenth/a quarter compared to the 1D-LC, likely due to the lack of separation and the resulting ion suppression. The dilution and the concentration of 2D-LC fractions prior to reinjection did not result in significant changes in feature counts (D, 3069/2777; E, 2795/3156). The fact that dried and reconstituted reinjections stood out with noticeably higher feature numbers (4001/3440) may again point to degradation and artifact formation caused by offline fraction preparation. The results of serial coupling LC-MS (768/45) were far below expectations. The reason is most probably to find in an inapt instrumental hardware setup. As the pressure limit of the mixed-mode column would have been highly exceeded by the common implementation, a third pump was used to mediate the flow from the first to the second column. However, feeding the split eluate of the first column through the volume of a pump head resulted in severe back-mixing and peak deformation before entering the second column. Thus, the hardware in hand did not facilitate the realization of serial column coupling with reasonable chromatographic performance.

**Fig. 7:**
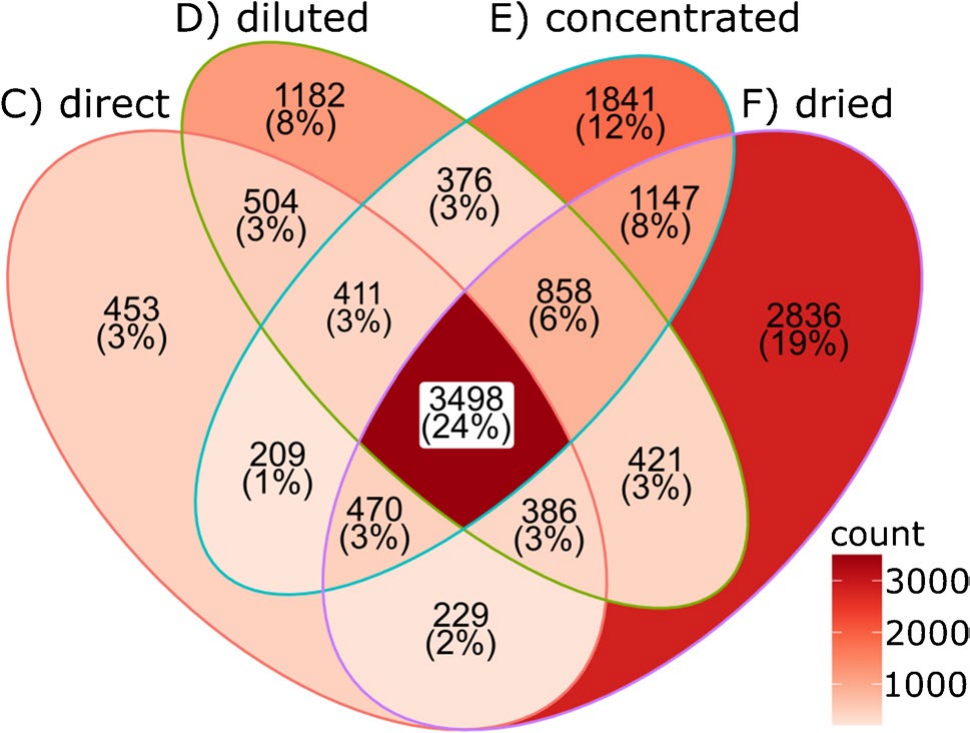
Venn diagram of individual features detected by offline 2D RP/IEX×HILIC separations with different fraction preparation procedures

In summary, the advantage of the offline 2D-LC approaches over other LC strategies is clearly established based on the detected feature numbers. Worth mentioning are the different practical dilution factors of approaches A to F. The complete sample was used for MS detection with DFI and 1D-LC, while in offline 2D-LC, partial reinjection of fractions resulted in around 20-fold (direct, diluted) and sevenfold (concentrated, dried) dilution. The untargeted, highly sensitive MS detection benefited more from the reduction of simultaneously arriving compounds in the ion source due to the additional separation dimension than from the undiluted sample introduction. MS signal intensity might be affected by the dilutions, but the consistency of results by C/D with E (threefold concentration) suggests that small dilution factors do not have a substantial effect on qualitative MS results. Offline fraction treatments imply a significant effort in lab work and bear several risks for contamination, precipitation, degradation, incomplete dissolution, or adhesion to vessel walls. No substantial gain in the number of detectable features was obtained by approaches D and E compared to C, and the increase from E to F hinted at artifact formation by the drying process. The Venn diagram in Fig. 7 illustrates the overlap of features detected in both ESI modes by the different 2D-LC fraction treatment approaches. The largest percentage (24%) of features was detected in all four experiments. The overlap of only two and three approaches in most cases was moderate with 1–6%, except for the cross-section of concentrated and dried fractions (8%). The number of features detected by the direct reinjection only was low (3%) and significantly higher for diluted (8%) and concentrated (12%) reinjection. Again, the dried reinjection stood out, with 19% of features exclusively detected with that procedure, which appears as another indication of artifact formation related to the complete drying process.

Eventually, we concluded that the complete evaporation and re-take-up of fractions was the least favorable approach in our study due to the risk of chemical alterations and significant manual effort. The concentration approach required similar effort but did result in only minor changes in detectable feature counts. The dilution of fractions with weak eluents to enable the reinjection of higher volumes is less complex and bears the potential for easy automation (e.g., by user-defined autosampler programs). In this study, it resulted in a similar outcome as the direct reinjection of fractions, which allows for fully automatic processing of the 2D-LC sequence.

Thus, we conclude that a direct reinjection of 5 µL of the collected native ^1^D fractions is the most efficient workflow in our offline RP/IEX×HILIC-MS approach regarding feature counts, fraction stability, and experimental effort. It will be the method of choice for the application in a comparative untargeted human urine profiling study of different groups to identify metabolomic markers for, e.g., coffee consumption.

## Conclusion

LC-MS plays a key role in the targeted and untargeted profiling of metabolites from biological materials, but the selection of the best-suited LC method is not an easy task due to highly diverse compound properties. To increase the metabolome coverage as much as possible, various techniques like dual, parallel, or serial combinations of RP and HILIC have been investigated in the past. The application of LC×LC is not popular in regular metabolomics studies yet. However, our previous study demonstrated the outstanding distribution of urine compounds in a 2D separation space spanned by a mixed RP/IEX mode and a HILIC dimension, making this combination highly attractive for untargeted compound detection. In our RP/IEX×HILIC-HRMS setup, we aimed for an offline fraction transfer from the ^1^D to the ^2^D to account for the inverted elution strengths of aqueous and organic mobile phases in the two modes and the risk of a significant resolution loss with an online approach. Injection experiments with a selection of target compounds showed that a 5 µL injection volume (corresponding to 1.5% of the ^2^D column volume) into the HILIC dimension was the best compromise in peak shape effects over a range of injection solvents from 0 to 100% water. Besides the independence of dimension run times, another essential benefit of offline LC×LC is the possibility of applying fraction preparation procedures to increase sensitivity. For a range of target compounds, the quantitative gains obtained by dilution with weak eluents to allow higher injection volumes, concentration by evaporation to one-third of the volume, and concentration to dryness and redissolving in one-third of the volume were not compelling. In fact, some compounds in evaporated fractions did even show decreased peak areas compared to untreated ones, hinting at some analyte loss or degradation. The evaluation of detectable unique feature counts from a urine sample verified the great potential of offline RP/IEX×HILIC-TOF-MS in untargeted compound detection compared to DFI and 1D-LC. Results from serial coupling were not applicable due to an inapt hardware setup. The different fraction preparation procedures did not significantly affect the feature counts in 2D-LC, except for the drying approach, which pointed again to some artifact formation. The overlap of detected features over the preparation procedures was high, but the concentration and especially drying procedures resulted in high numbers of exclusively detected features. For the sake of fraction stability, analysis time, and lab effort, the direct transfer of 5 µL untreated fractions will be the method of choice for future profiling studies of human urine.

## Supplementary Information

Below is the link to the electronic supplementary material.

Supplementary Material 1 (DOCX 386 KB)

## Data Availability

The datasets can be made available upon request from Andreas Dunkel.

## References

[1] Spagou K, Tsoukali H, Raikos N, Gika H, Wilson ID, Theodoridis G. Hydrophilic interaction chromatography coupled to MS for metabonomic/metabolomic studies. J Sep Sci. 2010;33:716–27. 10.1002/jssc.200900803. 20187037 10.1002/jssc.200900803

[2] Wolfender J-L, Marti G, Thomas A, Bertrand S. Current approaches and challenges for the metabolite profiling of complex natural extracts. J Chromatogr A. 2015;1382:136–64. 10.1016/j.chroma.2014.10.091. 25464997 10.1016/j.chroma.2014.10.091

[3] Wishart DS, Guo A, Oler E, Wang F, Anjum A, Peters H, Dizon R, Sayeeda Z, Tian S, Lee BL, Berjanskii M, Mah R, Yamamoto M, Jovel J, Torres-Calzada C, Hiebert-Giesbrecht M, Lui VW, Varshavi D, Varshavi D, Allen D, Arndt D, Khetarpal N, Sivakumaran A, Harford K, Sanford S, Yee K, Cao X, Budinski Z, Liigand J, Zhang L, Zheng J, Mandal R, Karu N, Dambrova M, Schiöth HB, Greiner R, Gautam V. HMDB 5.0: the human metabolome database for 2022. Nucleic Acids Res. 2022;50(D1):D622–31. 10.1093/nar/gkab1062. 34986597 10.1093/nar/gkab1062 PMC8728138

[4] Roca M, Alcoriza MI, Garcia-Cañaveras JC, Lahoz A. Reviewing the metabolome coverage provided by LC-MS: focus on sample preparation and chromatography-a tutorial. Anal Chim Acta. 2021;1147:38–55. 10.1016/j.aca.2020.12.025. 33485584 10.1016/j.aca.2020.12.025

[5] Ortmayr K, Causon TJ, Hann S, Koellensperger G. Increasing selectivity and coverage in LC-MS based metabolome analysis. TrAC Trends Anal Chem. 2016;82:358–66. 10.1016/j.trac.2016.06.011.

[6] Perez de Souza L, Alseekh S, Scossa F, Fernie AR. Ultra-high-performance liquid chromatography high-resolution mass spectrometry variants for metabolomics research. Nat Methods. 2021;18:733–46. 10.1038/s41592-021-01116-4. 33972782 10.1038/s41592-021-01116-4

[7] Harrieder E-M, Kretschmer F, Böcker S, Witting M. Current state-of-the-art of separation methods used in LC-MS based metabolomics and lipidomics. J Chromatogr B. 2022;1188: 123069. 10.1016/j.jchromb.2021.123069. 10.1016/j.jchromb.2021.123069 34879285

[8] Zhang T, Creek DJ, Barrett MP, Blackburn G, Watson DG. Evaluation of coupling reversed phase, aqueous normal phase, and hydrophilic interaction liquid chromatography with Orbitrap mass spectrometry for metabolomic studies of human urine. Anal Chem. 2012;84:1994–2001. 10.1021/ac2030738. 22409530 10.1021/ac2030738

[9] Lioupi A, Marinaki M, Virgiliou C, Begou O, Gika H, Wilson I, Theodoridis G. Probing the polar metabolome by UHPLC-MS. TrAC Trends Anal Chem. 2023;161: 117014. 10.1016/j.trac.2023.117014.

[10] Haggarty J, Burgess KEV. Recent advances in liquid and gas chromatography methodology for extending coverage of the metabolome. Curr Opin Biotechnol. 2017;43:77–85. 10.1016/j.copbio.2016.09.006. 27771607 10.1016/j.copbio.2016.09.006

[11] Ammann AA, Suter MJ-F. Multimode gradient high performance liquid chromatography mass spectrometry method applicable to metabolomics and environmental monitoring. J Chromatogr A. 2016;1456:145–51. 10.1016/j.chroma.2016.06.001. 27324626 10.1016/j.chroma.2016.06.001

[12] Grübner M, Dunkel A, Steiner F, Hofmann T. Systematic evaluation of liquid chromatography (LC) column combinations for application in two-dimensional LC metabolomic studies. Anal Chem. 2021;93:12565–73. 10.1021/acs.analchem.1c01857. 34491041 10.1021/acs.analchem.1c01857

[13] Yin P, Wan D, Zhao C, Chen J, Zhao X, Wang W, Lu X, Yang S, Gu J, Xu G. A metabonomic study of hepatitis B-induced liver cirrhosis and hepatocellular carcinoma by using RP-LC and HILIC coupled with mass spectrometry. Mol Biosyst. 2009;5:868–76. 10.1039/b820224a. 19603122 10.1039/b820224a

[14] Klavins K, Drexler H, Hann S, Koellensperger G. Quantitative metabolite profiling utilizing parallel column analysis for simultaneous reversed-phase and hydrophilic interaction liquid chromatography separations combined with tandem mass spectrometry. Anal Chem. 2014;86:4145–50. 10.1021/ac5003454. 24678888 10.1021/ac5003454

[15] Wang Y, Lehmann R, Lu X, Zhao X, Xu G. Novel, fully automatic hydrophilic interaction/reversed-phase column-switching high-performance liquid chromatographic system for the complementary analysis of polar and apolar compounds in complex samples. J Chromatogr A. 2008;1204:28–34. 10.1016/j.chroma.2008.07.010. 18692192 10.1016/j.chroma.2008.07.010

[16] Greco G, Grosse S, Letzel T. Serial coupling of reversed-phase and zwitterionic hydrophilic interaction LC/MS for the analysis of polar and nonpolar phenols in wine. J Sep Sci. 2013;36:1379–88. 10.1002/jssc.201200920. 23505207 10.1002/jssc.201200920

[17] Haggarty J, Oppermann M, Dalby MJ, Burchmore RJ, Cook K, Weidt S, Burgess KEV. Serially coupling hydrophobic interaction and reversed-phase chromatography with simultaneous gradients provides greater coverage of the metabolome. Metabolomics. 2015;11:1465–70. 10.1007/s11306-014-0770-7. 26366140 10.1007/s11306-014-0770-7 PMC4559102

[18] Chalcraft KR, McCarry BE. Tandem LC columns for the simultaneous retention of polar and nonpolar molecules in comprehensive metabolomics analysis. J Sep Sci. 2013;36:3478–85. 10.1002/jssc.201300779. 24030891 10.1002/jssc.201300779

[19] Pérez-Cova M, Tauler R, Jaumot J. Two-dimensional liquid chromatography in metabolomics and lipidomics. In: Wood PL, editor. Metabolomics. US, New York, NY: Springer; 2021. pp. 25–47. 10.1007/978-1-0716-0864-7_3.

[20] Fairchild JN, Horvath K, Gooding JR, Campagna SR, Guiochon G. Two-dimensional liquid chromatography/mass spectrometry/mass spectrometry separation of water-soluble metabolites. J Chromatogr A. 2010;1217:8161–6. 10.1016/j.chroma.2010.10.068. 21094946 10.1016/j.chroma.2010.10.068

[21] Willmann L, Erbes T, Krieger S, Trafkowski J, Rodamer M, Kammerer B. Metabolome analysis via comprehensive two-dimensional liquid chromatography: identification of modified nucleosides from RNA metabolism. Anal Bioanal Chem. 2015;407:3555–66. 10.1007/s00216-015-8516-6. 25736241 10.1007/s00216-015-8516-6

[22] Lv W, Shi X, Wang S, Xu G. Multidimensional liquid chromatography-mass spectrometry for metabolomic and lipidomic analyses. TrAC Trends Anal Chem. 2019;120: 115302. 10.1016/j.trac.2018.11.001.

[23] Wang S, Li J, Shi X, Qiao L, Lu X, Xu G. A novel stop-flow two-dimensional liquid chromatography-mass spectrometry method for lipid analysis. J Chromatogr A. 2013;1321:65–72. 10.1016/j.chroma.2013.10.069. 24238711 10.1016/j.chroma.2013.10.069

[24] Dugo P, Fawzy N, Cichello F, Cacciola F, Donato P, Mondello L. Stop-flow comprehensive two-dimensional liquid chromatography combined with mass spectrometric detection for phospholipid analysis. J Chromatogr A. 2013;1278:46–53. 10.1016/j.chroma.2012.12.042. 23332739 10.1016/j.chroma.2012.12.042

[25] Guo K, Peng J, Zhou R, Li L. Ion-pairing reversed-phase liquid chromatography fractionation in combination with isotope labeling reversed-phase liquid chromatography-mass spectrometry for comprehensive metabolome profiling. J Chromatogr A. 2011;1218:3689–94. 10.1016/j.chroma.2011.04.024. 21543078 10.1016/j.chroma.2011.04.024

[26] Navarro-Reig M, Jaumot J, Baglai A, Vivo-Truyols G, Schoenmakers PJ, Tauler R. Untargeted comprehensive two-dimensional liquid chromatography coupled with high-resolution mass spectrometry analysis of rice metabolome using multivariate curve resolution. Anal Chem. 2017;89:7675–83. 10.1021/acs.analchem.7b01648. 28643516 10.1021/acs.analchem.7b01648

[27] Montero L, Ibáñez E, Russo M, di Sanzo R, Rastrelli L, Piccinelli AL, Celano R, Cifuentes A, Herrero M. Metabolite profiling of licorice (*Glycyrrhiza glabra*) from different locations using comprehensive two-dimensional liquid chromatography coupled to diode array and tandem mass spectrometry detection. Anal Chim Acta. 2016;913:145–59. 10.1016/j.aca.2016.01.040. 26944999 10.1016/j.aca.2016.01.040

[28] van den Hurk RS, Pursch M, Stoll DR, Pirok BWJ. Recent trends in two-dimensional liquid chromatography. TrAC-Trend Anal Chem. 2023;166: 117166. 10.1016/j.trac.2023.117166.

[29] Lang R, Dieminger N, Beusch A, Lee Y-M, Dunkel A, Suess B, Skurk T, Wahl A, Hauner H, Hofmann T. Bioappearance and pharmacokinetics of bioactives upon coffee consumption. Anal Bioanal Chem. 2013;405:8487–503. 10.1007/s00216-013-7288-0. 23982107 10.1007/s00216-013-7288-0

[30] Chambers MC, Maclean B, Burke R, Amodei D, Ruderman DL, Neumann S, Gatto L, Fischer B, Pratt B, Egertson J, Hoff K, Kessner D, Tasman N, Shulman N, Frewen B, Baker TA, Brusniak M-Y, Paulse C, Creasy D, Flashner L, Kani K, Moulding C, Seymour SL, Nuwaysir LM, Lefebvre B, Kuhlmann F, Roark J, Rainer P, Detlev S, Hemenway T, Huhmer A, Langridge J, Connolly B, Chadick T, Holly K, Eckels J, Deutsch EW, Moritz RL, Katz JE, Agus DB, MacCoss M, Tabb DL, Mallick P. A cross-platform toolkit for mass spectrometry and proteomics. Nat Biotechnol. 2012;30:918–20. 10.1038/nbt.2377. 23051804 10.1038/nbt.2377 PMC3471674

[31] Smith CA, Want EJ, O’Maille G, Abagyan R, Siuzdak G. XCMS: processing mass spectrometry data for metabolite profiling using nonlinear peak alignment, matching, and identification. Anal Chem. 2006;78:779–87. 10.1021/ac051437y. 16448051 10.1021/ac051437y

[32] Tautenhahn R, Bottcher C, Neumann S. Highly sensitive feature detection for high resolution LC/MS. BMC Bioinformatics. 2008;9: 504. 10.1186/1471-2105-9-504. 19040729 10.1186/1471-2105-9-504 PMC2639432

[33] Benton HP, Want EJ, Ebbels TMD. Correction of mass calibration gaps in liquid chromatography–mass spectrometry metabolomics data. Bioinformatics. 2010;26:2488–9. 10.1093/bioinformatics/btq441. 20671148 10.1093/bioinformatics/btq441

[34] Prince JT, Marcotte EM. Chromatographic alignment of ESI-LC-MS proteomics data sets by ordered bijective interpolated warping. Anal Chem. 2006;78:6140–52. 10.1021/ac0605344. 16944896 10.1021/ac0605344

[35] Kolde R. _pheatmap: Pretty Heatmaps_. R package version 1.0.12. 2022; 10.32614/CRAN.package.pheatmap

[36] Gao C, Chen C, Akyol T, Dusa A, Yu G, Cao B, Cai P. ggVennDiagram: intuitive Venn diagram software extended. iMeta. 2024;3:1. 10.1002/imt2.177. 10.1002/imt2.177 PMC10989133 38868514

[37] D’Attoma A, Heinisch S. On-line comprehensive two dimensional separations of charged compounds using reversed-phase high performance liquid chromatography and hydrophilic interaction chromatography. Part II: application to the separation of peptides. J Chromatogr A. 2013;1306:27–36. 10.1016/j.chroma.2013.07.048. 23891372 10.1016/j.chroma.2013.07.048

[38] Fu L, Ding H, Han L, Jia L, Yang W, Zhang C, Hu Y, Zuo T, Gao X, Guo D. Simultaneously targeted and untargeted multicomponent characterization of Erzhi Pill by offline two-dimensional liquid chromatography/quadrupole-Orbitrap mass spectrometry. J Chromatogr A. 2019;1584:87–96. 10.1016/j.chroma.2018.11.024. 30473109 10.1016/j.chroma.2018.11.024

[39] Sun W, Tong L, Miao J, Huang J, Li D, Li Y, Xiao H, Sun H, Bi K. Separation and analysis of phenolic acids from *Salvia miltiorrhiza* and its related preparations by off-line two-dimensional hydrophilic interaction chromatography×reversed-phase liquid chromatography coupled with ion trap time-of-flight mass spectrometry. J Chromatogr A. 2016;1431:79–88. 10.1016/j.chroma.2015.12.038. 26792448 10.1016/j.chroma.2015.12.038

[40] Stoll DR, Talus ES, Harmes DC, Zhang K. Evaluation of detection sensitivity in comprehensive two-dimensional liquid chromatography separations of an active pharmaceutical ingredient and its degradants. Anal Bioanal Chem. 2015;407:265–77. 10.1007/s00216-014-8036-9. 25064601 10.1007/s00216-014-8036-9

[41] Schoonen J-W, Vulto P, Roo N, van Duynhoven J, van der Linden H, Hankemeier T. Solvent exchange module for LC-NMR hyphenation using machine vision-controlled droplet evaporation. Anal Chem. 2013;85:5734–9. 10.1021/ac401068j. 23679001 10.1021/ac401068j

[42] Tian H, Xu J, Guan Y. Comprehensive two-dimensional liquid chromatography (NPLC x RPLC) with vacuum-evaporation interface. J Sep Sci. 2008;31:1677–85. 10.1002/jssc.200700559. 18481322 10.1002/jssc.200700559

[43] Fornells E, Barnett B, Bailey M, Hilder EF, Shellie RA, Breadmore MC. Evaporative membrane modulation for comprehensive two-dimensional liquid chromatography. Anal Chim Acta. 2018;1000:303–9. 10.1016/j.aca.2017.11.053. 29289323 10.1016/j.aca.2017.11.053

[44] Vos J, Desmet G, Eeltink S. A generic approach to post-column refocusing in liquid chromatography. J Chromatogr A. 2014;1360:164–71. 10.1016/j.chroma.2014.07.072. 25127691 10.1016/j.chroma.2014.07.072

[45] Verstraeten M, Pursch M, Eckerle P, Luong J, Desmet G. Thermal modulation for multidimensional liquid chromatography separations using low-thermal-mass liquid chromatography (LC). Anal Chem. 2011;83:7053–60. 10.1021/ac201207t. 21815627 10.1021/ac201207t

[46] Van de Ven HCC, Gargano AFGFG, Van der Wal SJJ, Schoenmakers PJJ. Switching solvent and enhancing analyte concentrations in small effluent fractions using in-column focusing. J Chromatogr A. 2016;1427:90–5. 10.1016/j.chroma.2015.11.082. 26700154 10.1016/j.chroma.2015.11.082

[47] Chapel S, Heinisch S. Strategies to circumvent the solvent strength mismatch problem in online comprehensive two-dimensional liquid chromatography. J Sep Sci. 2022;45:7–26. 10.1002/jssc.202100534. 34525266 10.1002/jssc.202100534

[48] Chen Y, Montero L, Luo J, Li J, Schmitz OJ. Application of the new at-column dilution (ACD) modulator for the two-dimensional RP×HILIC analysis of *Buddleja davidii*. Anal Bioanal Chem. 2020;412:1483–95. 10.1007/s00216-020-02392-3. 31965244 10.1007/s00216-020-02392-3 PMC7026260

