# Supplementary material for: Comparative evaluation of comprehensive offline 2D-LC strategies coupled to MS for untargeted metabolomic studies of human urine
Source: Anal Bioanal Chem. 2025 Nov 1;417(30):7013–23. doi: 10.1007/s00216-025-06195-2 (PMC12680765; doi:10.1007/s00216-025-06195-2)
Supplement: Supplementary file 1 — Supplementary Material 1 (DOCX 386 KB) [file 216_2025_6195_MOESM1_ESM.pdf]

Supplementary Information

Comparative evaluation of comprehensive offline 2D-LC strategies coupled to MS for untargeted metabolomic studies of human urine

Maria Grübner[1,2], Andreas Dunkel[1,3], Frank Steiner[2], Thomas Hofmann[1]

[1] Chair of Food Chemistry and Molecular Sensory Science, Technical University of Munich, Lise-Meitner-Straße 34, 85354 Freising, Germany
[2] Thermo Fisher Scientific, Dornierstraße 4, 82110 Germering, Germany
[3] Institute of Physiological Chemistry, Faculty of Chemistry, University of Vienna, Josef-Holaubek-Platz 2, 1090 Vienna, Austria

SI-1: Test mixtures

Test mixture for HILIC injection experiments covering the HILIC gradient:
caffeine, uracil, 7-methylxanthine, 1,3-dimethyluric acid, allantoin, inosine, serotonine, pantothenic acid, mannitol, taurine, trigonelline, sarcosine, creatine, lactose, N-acetyl-aspartic acid, 5-aminopentanoic acid, carnitine, glutamic acid, putrescine

Test mixture for targeted fraction treatment comparison covering the mixed-mode RP/IEX gradient:
alanine, choline, proline, leucine, ornithine, urocanic acid, acetylcholine, glutamic acid, carnitine, phenylalanine, 2-phenylacetamide, hippuric acid, tyrosine, acetylcarnitine, tryptophan, melatonin, biotin, adenosine, octanoyl carnitine, testosterone, adenosine 5'-monophosphate, riboflavine, palmitoyl carnitine

SI-2: Instrumental setup for serial column coupling

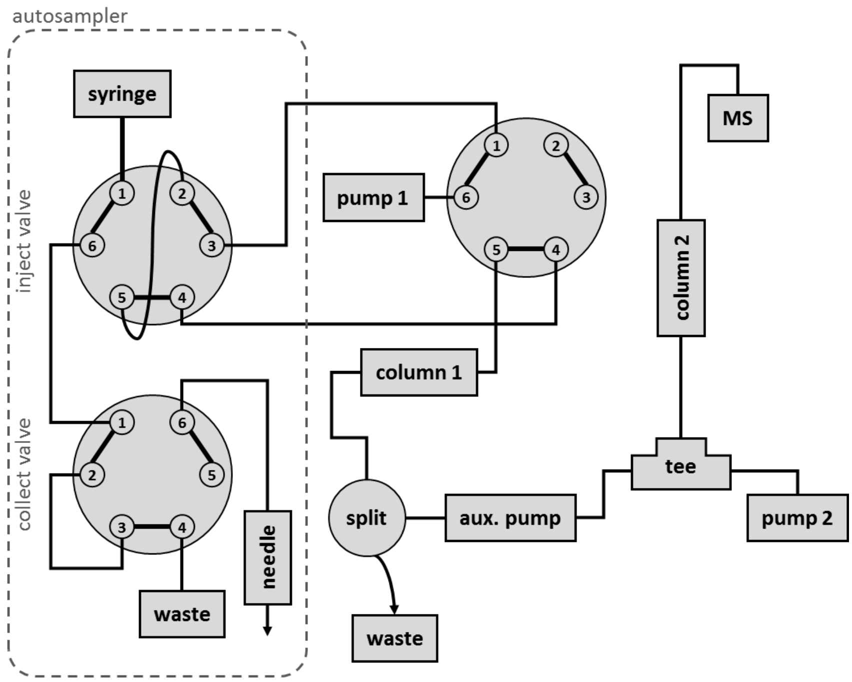

SI-3: Mixed-mode and HILIC gradients and MS conditions

- *Acclaim Trinity P1 column (50×2.1 mm, 3 µm, Thermo Fisher Scientific, Sunnyvale, USA)*: mobile phase A: acetonitrile, B: water, C: 200 mM NH_4Ac in water pH 3.8

MM-1	0.3mL/min		
min	A	B	C
0.0	0	97.5	2.5
1.0	0	95	5
5.0	20	55	25
10	75	0	25
15	75	0	25
16	0	97.5	2.5
20	0	97.5	2.5

MM-2	0.2mL/min		
min	A	B	C
0.0	0	97.5	2.5
6.5	20	55	25
8.5	20	55	25
14.5	75	0	25
16.5	75	0	25
18.5	97.5	0	2.5
22.5	97.5	0	2.5
23.0	0	97.5	2.5
28	0	97.5	2.5

- *Accucore-150-Amide-HILIC column (150×2.1 mm, 2.6 µm, Thermo Fisher Scientific, Sunnyvale, USA)*: mobile phase A: ACN, B: water, C: 100 mM NH_4Ac in water pH 4.3

H-1	0.4mL/min		
min	A	B	C
0.0	90	0	10
6.0	60	30	10
8.0	20	70	10
10.0	20	70	10
11.0	90	0	10
15.0	90	0	10

microTOF-Q conditions with gradient H-1:
positive ESI; full scan 50-1000 m/z; capillary voltage -4500 V; end plate offset -500 V; nitrogen as drying and nebulizing gas 180 °C/ 1.6 bar/ 10 L/min; quadrupole ion energy 5 eV

H-2	0.4mL/min		
min	A	B	C
0.0	90	0	10
6.0	60	30	10
8.0	20	70	10
9.0	20	70	10
10.0	90	0	10
14.0	90	0	10

API 3200 triple quadrupole MS conditions with gradient H-2:
positive ESI; multiple reaction monitoring (MRM) mode; ion spray voltage 5500 V; temperature 450 °C; quadrupoles at unit mass resolution; nitrogen as nebulizer gas 55 psi, turbo gas 65 psi, curtain gas 25 psi and collision gas
Mass transitions of the parent ions [M]+ or [M+H]+ into specific product ions induced by collision-induced dissociation (CID, dwell time 10 ms) were recorded. For tuning each analyte was constantly infused by a syringe pump (10 µL/min).

H-3	0.4mL/min		
min	A	B	C
0.0	90	0	10
0.5	90	0	10
6.5	60	30	10
9.5	15	75	10
12.0	15	75	10
12.5	90	0	10
17.0	90	0	10

TripleTOF 6600 conditions with gradient H-3:
All experiments were recorded in positive and in negative, high sensitivity ESI mode with TOF-MS full scan followed by information-dependent (IDA) product ion acquisition in a range of 50-1000 m/z. Source parameters were: temperature 500 °C, ion spray voltage 5500 V and -4500 V, curtain gas 35 psi, nebulizer gas 55 psi, heater gas 65 psi, declustering potential 80 V and -80 V. Accumulation time was 250 ms for TOF-MS scan and 10 ms for MS/MS scan. At a max 8 candidate ions were monitored, exceeding a threshold of 100 cps with dynamic background subtraction under exclusion of isotopes within 4 Da. Collision energy spread was used with 35± 20 V in positive mode and -35 V ± 20 V in negative mode. After each tenth injection the instruments calibration was verified and corrected using ESI Positive or ESI Negative Calibration solution (Sciex) and a Calibrant Delivery System (Sciex)

H-4	0.35mL/min		
min	A	B	C
0.0	100	0	0
4.0	100	0	0
16.0	65	35	0
22.0	15	85	0
25.0	15	85	0
25.5	100	0	0
30.0	100	0	0

The effective solvent composition in the HILIC column was a combination of 0.05 ml/min eluate from mixed-mode column gradient MM-2 with the gradient H-4. Same MS conditions applied as with gradient H-3.

SI-4: Comparison of fraction preparation procedures

Table SI-1. Peak area ratios related to directly reinjected fractions

Analyte	Fraction	direct (4 µL)	diluted (10 µL)	concentrated (4 µL)	dried (4 µL)
alanine	1	1.00	1.36	2.53	2.70
choline	7	1.00	1.01	1.34	1.90
proline	1	1.00	1.37	2.38	2.52
leucine	1	1.00	1.44	2.84	3.03
ornithine	11	1.00	1.33	2.54	2.53
urocanic acid	8	1.00	1.21	1.95	1.83
acetylcholine	7	1.00	1.18	1.43	1.33
glutamic acid	3	1.00	1.42	4.41	4.19
carnitine	4	1.00	1.11	1.43	1.43
phenylalanine	1	1.00	1.43	2.61	2.62
2-phenylacetamide	2	1.00	1.15	2.25	2.16
hippuric acid	15	1.00	1.32	2.26	1.95
tyrosine	1	1.00	1.44	2.93	2.75
acetylcarnitine	3	1.00	1.11	1.52	1.44
tryptophan	7	1.00	1.93	6.30	5.91
melatonin	9	1.00	1.13	1.91	1.75
biotin	9	1.00	1.22	2.80	1.68
adenosine	6	1.00	1.18	1.75	1.39
octanoylcarnitine	10	1.00	1.20	1.53	1.19
testosterone	15	1.00	1.07	1.74	1.69
AMP	11	1.00	1.09	2.94	3.78
riboflavin	4	1.00	1.19	1.66	0.96
palmitoylcarnitine	18	1.00	1.36	0.47	1.64
**mean**		**1.00**	**1.27**	**2.33**	**2.28**

SI-5: Numbers of unique features detected by the different LC approaches coupled to TOF-MS

		feature counts	
code	LC-MS approach	ESI positive	ESI negative
A	DFI	119	134
B	1D-LC	1350	476
C	2D-LC direct	2864	2598
D	2D-LC diluted	3069	2777
E	2D-LC conc.	2795	3156
F	2D-LC dried	4001	3440
G	serial coupling	768	45
